# Pixel-Based Long-Wave Infrared Spectral Image Reconstruction Using a Hierarchical Spectral Transformer

**DOI:** 10.3390/s24237658

**Published:** 2024-11-29

**Authors:** Zi Wang, Yang Yang, Liyin Yuan, Chunlai Li, Jianyu Wang

**Affiliations:** 1 Key Laboratory of Space Active Opto-Electronics Technology, Shanghai Institute of Technical Physics, Chinese Academy of Sciences, Shanghai 200083, China; wangzi@shanghaitech.edu.cn (Z.W.); yangyang@mail.sitp.ac.cn (Y.Y.); yuanliyin@mail.sitp.ac.cn (L.Y.); 2 University of Chinese Academy of Sciences, Beijing 100049, China; 3 School of Information Science and Technology, ShanghaiTech University, Shanghai 201210, China; 4 Hangzhou Institute for Advanced Study, University of Chinese Academy of Sciences, Hangzhou 310024, China

**Keywords:** spectral reconstruction, long-wave infrared, thermal infrared, spectral imaging, deep learning, transformer, infrared imaging spectrometer

## Abstract

Long-wave infrared (LWIR) spectral imaging plays a critical role in various applications such as gas monitoring, mineral exploration, and fire detection. Recent advancements in computational spectral imaging, powered by advanced algorithms, have enabled the acquisition of high-quality spectral images in real time, such as with the Uncooled Snapshot Infrared Spectrometer (USIRS). However, the USIRS system faces challenges, particularly a low spectral resolution and large amount of data noise, which can degrade the image quality. Deep learning has emerged as a promising solution to these challenges, as it is particularly effective at handling noisy data and has demonstrated significant success in hyperspectral imaging tasks. Nevertheless, the application of deep learning in LWIR imaging is hindered by the severe scarcity of long-wave hyperspectral image data, which limits the training of robust models. Moreover, existing networks that rely on convolutional layers or attention mechanisms struggle to effectively capture both local and global spectral correlations. To address these limitations, we propose the pixel-based Hierarchical Spectral Transformer (HST), a novel deep learning architecture that learns from publicly available single-pixel long-wave infrared spectral databases. The HST is designed to achieve a high spectral resolution for LWIR spectral image reconstruction, enhancing both the local and global contextual understanding of the spectral data. We evaluated the performance of the proposed method on both simulated and real-world LWIR data, demonstrating the robustness and effectiveness of the HST in improving the spectral resolution and mitigating noise, even with limited data.

## 1. Introduction

Long-wave infrared (LWIR) or thermal infrared refers to wavelengths ranging between 7 and 14 μm. A range of crucial materials, including minerals and gases, have distinctive spectral characteristics within this LWIR range, enabling their identification [1]. Consequently, LWIR spectral imaging plays a crucial role in various applications such as environmental monitoring, climate studies, gas monitoring, mineral exploration, and fire detection [2,3].

In recent years, the computational spectral imaging technique, powered by advanced algorithms, has enabled the acquisition of high-quality spectral images in real time [4]. A prime example is the recently proposed Uncooled Snapshot Infrared Spectrometer (USIRS), which has enabled real-time video imaging in the LWIR range [5,6]. This is achieved by replicating the target scene using optical multi-aperture imaging and assigning filters of different wavelengths for each scene [7], allowing for real-time LWIR spectral video imaging at 8Hz. However, the USIRS presents two significant challenges. Firstly, the spectral resolution is achieved via the band-pass spectral filtering of the multi-aperture scene, creating a trade-off between the spectral and spatial resolution. To preserve an adequate spatial resolution, the spectral resolution is kept relatively low, limiting the USIRS to only 18 spectral channels. Secondly, the uncooled LWIR system, unlike commercial visible cameras that measure reflected sunlight, measures the target scene’s inherent radiation, typically low-intensity blackbody radiation at room temperature. This radiation is highly sensitive to background noise, resulting in a low signal-to-noise ratio (SNR) [8,9]. This further degrades the quality of spectrum acquisition and imaging performance. Therefore, a specifically designed computational algorithm is required to address these challenges.

Over the past decade, deep learning has demonstrated extraordinary robustness to noise [10,11] and high accuracy in hyperspectral image reconstruction and spectroscopy reconstruction [12], making it a promising solution to the challenges faced by USIRS. This method successfully integrates both spatial and spectral information to reduce noise and preserve details in hyperspectral images, leading to significant improvements in image quality [13,14,15,16]. Liu et al. advanced hyperspectral image denoising using a 3D attention denoising network [17]. By incorporating a three-dimensional attention mechanism, they managed to effectively capture intricate patterns and relationships within the hyperspectral data, resulting in superior denoising performance. Zhang et al. enhanced hyperspectral image denoising using a multitask learning approach combined with sparse representation techniques [18].

On the other hand, the field of spectroscopy reconstruction has introduced many pixel-based spectrum reconstruction methods, which mitigate the need for a large number of spectral images to train a neural network [19,20,21,22]. For instance, Wen et al. utilized the Multilayer Perceptron (MLP) to efficiently reconstruct the absolute spectra of various nonluminous samples, thereby developing a miniaturized spectrometer [23]. J. Zhang et al. suggested feeding the initial results of optimization-based methods into the MLP instead of directly feeding the raw measurements [24]. This reconstruction method has shown great potential for spectral recovery of filter-based miniature spectrometers. However, in the task of LWIR spectral reconstruction, existing neural network structures struggle to handle the noise and unique characteristics of the LWIR spectrum. LWIR data can be noisy, requiring neural networks to be robust against such noise to avoid inaccurate reconstructions. Traditional Convolutional Neural Networks (CNNs) [25,26,27,28] often struggle with this task due to their limited receptive fields and inefficient modeling of long-range dependencies [29]. Furthermore, the reconstruction problem is inherently ill posed, indicating that multiple possible solutions often exist for a given set of measurements. This complexity makes it challenging for MLPs [30,31,32] to learn meaningful representations without significant computational overhead and a risk of overfitting [33]. Therefore, there is a demand for bespoke network architectures for LWIR spectral reconstruction. These should be capable of capturing the complex spectral correlations and dependencies that are inherent to the LWIR spectrum.

To address these challenges, we propose the pixel-based Hierarchical Spectral Transformer (HST). First, instead of directly training on LWIR spectral images, we train the pixel-based neural network on public LWIR spectral databases [34]. This strategy reduces the need for high-quality paired data for neural network training. Second, we incorporate an LWIR spectral noise model into the USIRS data simulation pipeline. The noise model serves as a bridge between synthetic data and experimental data, enabling our network, trained on synthetic data, to effectively handle experimental data that are collected by the USIRS. Third, the HST employs a multistage architecture, where each stage progressively refines the spectral features, enhancing the detail and accuracy of the reconstruction. By employing spectral-wise multi-head self-attention [35], the HST can concentrate on the most relevant spectral features, thereby reducing the computational complexity and enhancing the reconstruction quality. This hierarchical approach ensures that both global and local spectral information effectively mitigate the large amount of noise and achieve superior performance in LWIR spectral image reconstruction tasks. Furthermore, while the HST is specifically designed and evaluated for the USIRS system, its versatility allows for it to be applied to other computational spectral imaging systems.

Our contributions are summarized as follows:

- We propose training the neural network on pixel-based LWIR spectra and integrating an LWIR spectral noise model into the USIRS data simulation pipeline. This approach reduces the need for high-quality paired data for training on LWIR spectral images.
- We introduce the Hierarchical Spectral Transformer (HST), designed to effectively learn and preserve both global and local spectral information, thus mitigating the large amount of noise and enhancing the reconstruction accuracy.
- We evaluate our pipeline using both synthetic and experimental data to demonstrate its effectiveness in handling real-world scenarios.

## 2. Materials and Methods

### 2.1. The USIRS

**Figure 1:**
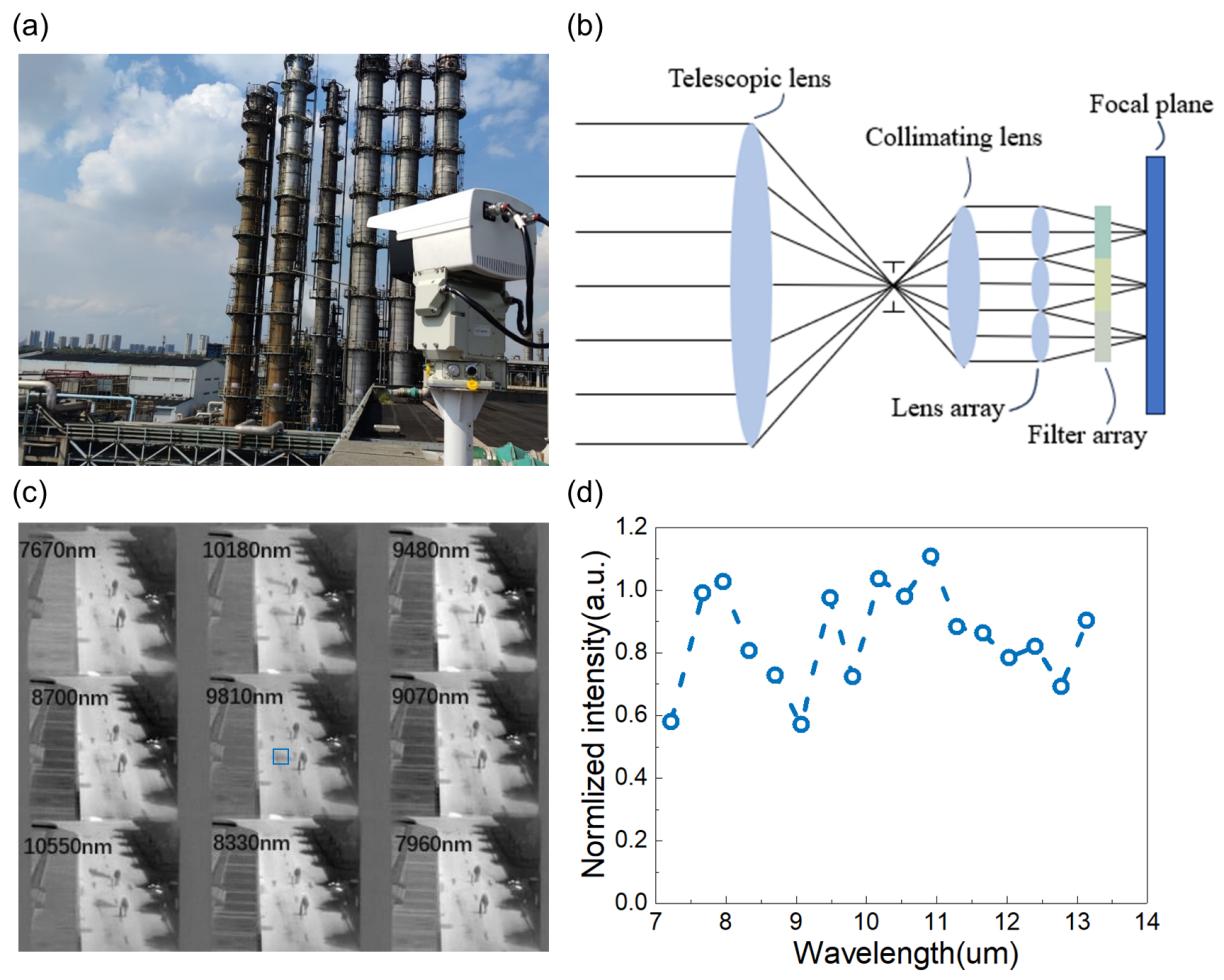
(**a**) Photograph of the USIRS for monitoring the gas emission in an industrial zone. (**b**) Optical schematic diagram of the USIRS. (**c**) Part of the captured raw data of the USIRS. (**d**) The captured noise intensity of the target gas in the blue zone.

The Uncooled Snapshot Infrared Spectrometer (USIRS) is an industrial instrument developed by the Shanghai Institute of Technical Physics of the Chinese Academy of Sciences, as shown in Figure 1a.

The USIRS uses filters and multi-aperture imaging to capture images at various wavelengths, providing a detailed spectral image of the target object. Figure 1b illustrates the optical design of the USIRS. The process begins with a telescopic lens collecting light from the target. This light is then directed to a collimating lens, ensuring that the rays are parallel. Following this, a lens array composed of nine smaller lenses recreates the target scene. The light then travels through a filter array, in which each filter corresponds to a specific waveband, before it finally reaches the focal plane, where images of different wavelengths are created and captured.

The USIRS utilizes two identical optical systems, with the only difference being the wavelength of the filter array. This effectively makes it a dual system with 17 band-pass wavelengths: 7.22 μm, 7.67 μm, 7.96 μm, 8.33 μm, 8.70 μm, 9.07 μm, 9.48 μm, 9.81 μm, 10.18 μm, 10.55 μm, 10.92 μm, 11.29 μm, 11.66 μm, 12.03 μm, 12.40 μm, 12.77 μm, and 13.14 μm.

Figure 1c displays one of the USIRS images for a target scenario involving two individuals and two gas bottles releasing NH3 and SF6, respectively. The spectral curves of the gases marked by the blue box are depicted in Figure 1d. The USIRS does not have a high spectral resolution, given that it only has 17 band-pass spectral channels and the measurement tends to be quite noisy. For further applications, it is crucial to denoise and reconstruct the high-resolution spectrum.

### 2.2. Imaging and Noise Model

Deep learning has emerged as a promising solution to the problem of hyperspectral reconstruction with large amounts of noise. However, the challenge in using neural networks for long-wave infrared spectral video reconstruction lies in the scarcity of sufficient high-quality data for training. Most existing long-wave infrared hyperspectral datasets consist of single-point spectral data collected through spectrometers. Therefore, we propose to develop the imaging and noise model of the USIRS, allowing us to generate corresponding low-resolution data from high-resolution ones using this model and subsequently train the neural network at the pixel spectrum level.

The signal received by the USIRS is a combination of the target radiation and background radiation:

(1) $$L\left(\lambda \right)=L_{t}\left(\lambda \right)\tau \left(\lambda \right)+L_{p}\left(\lambda \right)$$

Here, $L_{t}\left(\lambda \right)$ represents the radiation spectrum of the target on the lens of the USIRS. L(λ) is the radiation reaching the detector, τ(λ) is the spectral response of the imaging system, and $L_{p}\left(\lambda \right)$ is the background radiation. The radiation reaching the the detector is converted into a current and then quantified as a digital number (DN):

(2) $$DN=P\left(\phi L\right)+N_{d}+N_{q}$$

In this equation, P is the photon noise operator, ϕ is the quantum efficiency of the detector, $N_{d}$ is the dark current in the circuits, and $N_{q}$ is the quantization noise. Both the dark currents $N_{d}$ and $N_{q}$ quantization noise are assumed to obey the Gaussian distribution, with $N(\mu_{d},\sigma_{d})$ and $N(\mu_{q},\sigma_{q})$, respectively.

The imaging and noise model provides a pipeline for converting publicly available high-resolution spectral data into synthetic measurements of the USIRS, thus providing paired data for neural network training.

### 2.3. Pixel-Based Hierarchical Spectral Transformer

We propose the Hierarchical Spectral Transformer (HST), a model that utilizes self-attention to capture long-range dependencies and spectral correlations. This model employs a multistage architecture to progressively refine spectral and spatial features, thereby enhancing the reconstruction accuracy. This approach preserves both global and local spectral information, improving the performance in LWIR spectral image reconstruction tasks.

**Figure 2:**
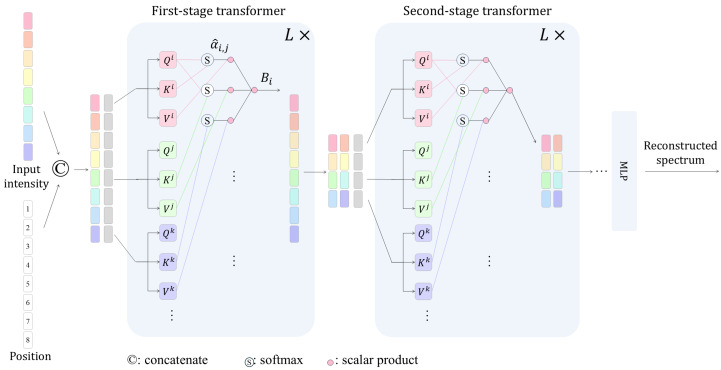
Schematic representation of the proposed HST. This network employs a point-based transformer to process the input spectral intensity by breaking it down into individual point tokens. These tokens are processed through several transformer blocks using multi-head self-attention. As the process advances, the merging of layers decrease the number of tokens while simultaneously doubling the channel depth, thereby creating an effective hierarchical representation.

The network structure of the proposed HST is illustrated in Figure 2.

#### 2.3.1. Positional Encoding

The input of the network is the measured intensity, denoted as $I\in R^{l}$ in different spectral channels, concatenated with the corresponding positional encoding, denoted as $Pos\in R^{2Ml}$. Each feature’s position, denoted as *m*, is transformed into a positional embedding via a trigonometric transformation:

(3) $$\begin{array}{cc} Pos(m)= & [\sin\left(m\times 2^{0}\right),\cos\left(m\times 2^{0}\right),\\ & \;\sin\left(m\times 2^{1}\right),\cos\left(m\times 2^{1}\right),\\ & \;\ldots \\ & \;\sin\left(m\times 2^{M}\right),\cos\left(m\times 2^{M}\right)] \end{array}$$

In this equation, *M* is the maximum encoding dimension, set to 5 in our system. The combined input is as follows:

(4) $$S=I⊕Pos\in R^{(2M+1)l}$$

The combined input is fed into the multistage transformer. This process ensures that each position has a unique encoding, allowing the model to comprehend the order of the input data.

#### 2.3.2. Hierarchical Representation

We employ a point-based transformer to process the input intensity. In contrast to standard visual transformers that work with patches, this method divides the input spectral intensity into separate point tokens. These tokens are then processed using multiple transformer blocks, each integrating multi-head self-attention computations.

We construct a hierarchical representation by reducing the token count with the help of merging layers as we progress deeper into the stages. These merging layers combine the features of every two adjacent tokens and apply a linear layer to the combined features. This process results in halving the token count while doubling the channel depth. All these stages collectively yield a hierarchical representation. In the *k*-th stage, the output of the transformer is

(5) $$S^{\left(k\right)}=Transformer\left\{S^{\left(k-1\right)}\right\}$$

In this equation, $S^{\left(k\right)}\in R^{\left(2M+1\right)l/2^{k}}$ is the output of the *k*-th stage transformer, and $S^{\left(k-1\right)}\in R^{\left(2M+1\right)l/2^{\left(k-1\right)}}$ is the input of the *k*-th stage transformer. In addition, k=1,2,3,…. In the first stage, we define the input of the first stage as $S^{\left(k-1\right)}=S$.

In our experiments, we use a 5-stage representation to convert the 17 channels into a single channel but with a feature dimension of 32. In each stage, we repeat the transformer block L=4 times.

#### 2.3.3. Attention Mechanism in Transformer

Each stage employs multi-head attention to facilitate token reduction. The attention mechanism allows the model to focus on the most relevant parts of the input data by assigning different weights to different parts of the input.

In the transformer, the input of the *k*-th stage of the transformer $S^{\left(k-1\right)}$ is first linearly transformed by three learnable weight matrices, $W_{Q},\;W_{K},$ and $W_{V}$, to obtain the queries *Q*, keys *K*, and values *V*, respectively. We denote the queries at the *i*-th position as $Q_{i}$. And the keys and values follow the same rule. The weight from the *i*-th and *j*-th position is computed as follows:

(6) $${\hat{\alpha }}_{i,j}=\mathrm{softmax}\left(\frac{Q_{i}{K_{j}}^{T}}{h}\right)$$

where *h* is the length of the dimension of queries and keys. The attention at the *i*-th position is computed as follows:

(7) $$B_{i}=\sum_{j}{\hat{\alpha }}_{i,j}V_{j}$$

The output is a weighted sum of the values, allowing the model to focus on the most relevant parts of the input data. At the end of this stage, we concatenate the attention $B=\mathrm{concat}(B_{1},B_{2},\ldots ,B_{l})$ and further pass the matching features through a linear layer:

(8) $$S^{\left(k\right)}=\mathrm{linear}\left\{B\right\}$$

In each stage, the attention mechanism can be repeated L times to produce a deep neural network. In this case, the linear layer is applied only when the last attention mechanism outputs.

#### 2.3.4. Implementation Details

Our network training employs the Mean Squared Error (MSE) loss, which is mathematically defined as follows:

(9) $$MSE=\frac{1}{n}\sum_{i=1}^{n}{\left(s_{i}-{\hat{s}}_{i}\right)}^{2}$$

In this equation, *n* represents the total number of data points, $s_{i}$ is the ground truth spectrum, and ${\hat{s}}_{i}$ is the reconstructed spectrum. This formula calculates the average of the squared differences between the ground truth and predicted spectrum. Larger errors are penalized more heavily, pushing the model to reduce significant discrepancies and aim for more accurate predictions. The use of MSE loss enhances the precision and reliability of our network, ensuring consistent and reliable results.

We trained our network with a batch size of 100 for 1000 epochs. The Adam optimizer was used with an initial learning rate of 0.001, which decays by a factor of 0.5 every 100 epochs. The minimum learning rate was set to $1\times 10^{-5}$. The HST was implemented using PyTorch 1.8, and the training process took approximately 3 h on an NVIDIA 3090 GPU.

## 3. Results

We first evaluated the effectiveness of our method using synthetic data.

This synthetic dataset, employed for the evaluation, comprises four sections. The first section includes 1289 samples from the ASTER LWIR spectral library, selected for their distinctive LWIR attributes. The ASTER library is a comprehensive collection housing over 2300 spectra of natural and human-made materials. It covers a wide range of materials, and the wavelengths range from 0.4 to 15.4 µm. The library includes the spectra of various substances such as rocks, minerals, lunar soils, terrestrial soils, human-made materials, meteorites, vegetation, snow, and ice. The second section contains 1000 simulated samples of blackbody radiation, ranging from 100K to 1000K, in line with Planck’s law. The third section presents the gas emission spectra of 24 characteristic LWIR gases, including Methane (CH4), Ethylene (C2H4), ammonia (NH3), sulfur hexafluoride (SF6), Cyclohexane (C6H12), Methanol (CH3OH), Acetone (CH3COCH3), trimethylamine ((CH3)3N), Cyclopropane (C3H6), Propylene (C3H6), trans-2-butene (C4H8), Butadiene (C4H6), Ethylene oxide (C2H4O), Dimethylamine (HN(CH3)2), vinyl chloride (C2H3Cl), 1-Butene (C3H8), Acetylene (C2H2), Propyne (C3H4), Dimethyl ether (C2H6O), Acetaldehyde (CH3CHO), Chloroethane (C2H5Cl), Chloromethane (CH3Cl), Methylamine (CH3NH2), and Sulfur dioxide (SO_2_). Lastly, the fourth section simulates the radiation spectrum of gas in front of a blackbody, based on the model in [6], and includes 2400 samples.

**Figure 3:**
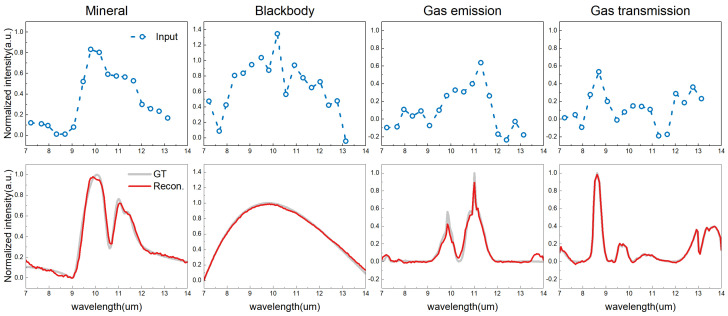
Qualitative evaluation of the proposed HST for spectrum reconstruction of four different types of spectra. The blue dashed lines in the top row represent the input intensity. The bottom row shows the ground truth in gray compared with the reconstructed spectrum in red. The reconstructed data closely match the ground truth across all types, highlighting the effectiveness of our proposed HST.

We began with a qualitative evaluation of the proposed HST’s ability to handle four different spectrum types—minerals, blackbody, gas emission, and gas transmission—and to generate high-resolution spectrum reconstruction, as depicted in Figure 3. The reconstructed data aligned closely with the ground truth across all types, highlighting the effectiveness of our reconstruction method. This figure emphasizes the importance of accurate spectral data replication in applications such as remote sensing, spectroscopy, and material identification.

For a quantitative evaluation of the reconstruction’s performance, we employed general metrics. The correlation coefficient, for example, provides insights into the linear relationship between two spectra:

(10) $$r=\sum_{i=1}^{l}\frac{\left(s_{i}-\bar{s}\right)\left(g_{i}-\bar{g}\right)}{l\sigma_{s}\sigma_{g}}$$

where *s* and *g* represent the target and ground truth spectra, respectively. $s_{i}$ and $g_{i}$ denote the sampled spectra indexed with *i*. $\bar{s}$ and $\bar{g}$ are the means of *s* and *g*, respectively. $\sigma_{s}$ and $\sigma_{g}$ represent the standard deviations of *s* and *g*, respectively. Lastly, *l* refers to the length of each spectrum.

The Root Mean Squared Error (RMSE) is another commonly used metric for comparing the differences between a reconstructed spectrum and the ground truth. The RMSE is the square root of the average of these differences squared. Using *s* and *g* as the target and ground truth spectra, respectively, the RMSE is defined as follows:

(11) $$RMSE=\sqrt{\frac{1}{l}\sum_{i=1}^{l}{\left(s_{i}-g_{i}\right)}^{2}}$$

where *l* is the length of each spectrum vector, $\sum_{i=1}^{l}$ is the sum from i=1 to *l*, $s_{i}$ is the sampled result for the reconstructed spectrum at index *i*, and $g_{i}$ is the sampled result for the ground truth at index *i*. ${\left(s_{i}-g_{i}\right)}^{2}$ is the squared difference between the spectrum and the ground truth at index *i*. The RMSE is always non-negative, and a lower value is generally more desirable.

The Peak Signal-to-Noise Ratio (PSNR) is a widely used metric in spectrum reconstruction for assessing the quality of a reconstructed spectrum in comparison to the ground truth [36]. Using *s* and *g* as the target and ground truth spectra, respectively, the PSNR is defined as follows:

(12) $$PSNR=20\cdot \log_{10}\left(\frac{MAX_{s}}{\sqrt{MSE}}\right)$$

where $MAX_{s}$ is the maximum possible spectrum intensity value. For a normalized spectrum, this is set to 1. MSE is the Mean Squared Error between the target and ground truth spectra and is defined as follows:

(13) $$MSE=\frac{1}{n}\sum_{i=1}^{n}{\left(s_{i}-g_{i}\right)}^{2}$$

where *n* is the length of the spectrum, and $s_{i}$ and $g_{i}$ are the intensity values of the reconstructed and ground truth spectra, respectively, at the *i*-th position. A higher PSNR indicates better image quality, indicating less distortion due to noise.

**Figure 4:**
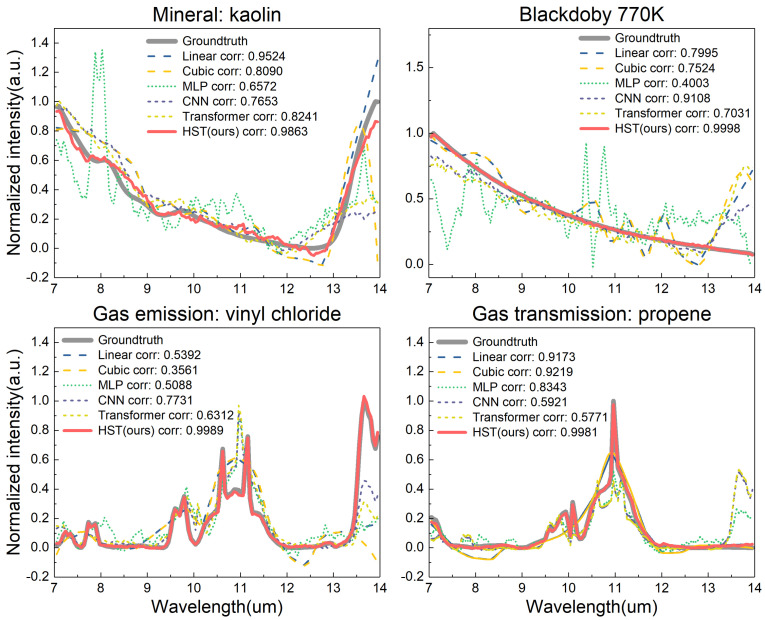
Comparison of different methods using synthetic datasets. We compared the proposed HST with other neural network methods and interpolation method for spectrum reconstruction for four materials: kaolin, a 770 K blackbody, vinyl chloride gas emission, and propene gas transmission. Each subplot presents the ground truth data (solid gray line) against predictions from six methods: Linear Interpolation (dashed blue line), Cubic Spline Interpolation (dashed yellow line), MLP (dotted green line), CNN (dotted cyan line), Transformer model (dotted orange line), and HST (solid red line). The correlation coefficient of the reconstructed spectrum with the ground truth is shown in the legend.

We quantitatively evaluated our proposed methods and compared them with various other methods. In Figure 4, our proposed HST is compared with other neural network methods and interpolation methods for spectrum reconstruction for four materials: kaolin, a 770 K blackbody, vinyl chloride gas emission, and propene gas transmission. The correlation coefficient of the reconstructed spectrum with the ground truth is indicated in the legend. Our proposed HST consistently demonstrated the highest fidelity to the ground truth across all materials. The Linear and Cubic methods offer simple approximations, while deep learning approaches—MLP [32], CNN [26], and Transformer [35]—demonstrate varying degrees of improvement, with the HST model achieving superior performance.

We further assessed the proposed HST and other methods at different noise levels on synthetic data, as shown in Table 1. The performances of various interpolation and neural network methods (Linear Interpolation, Cubic Interpolation, MLP, CNN, Transformer, and HST) in spectrum reconstruction using synthetic data across different noise levels (0, 0.1, 0.2, 0.5) were compared. Performance was measured using the Root Mean Squared Error (RMSE), correlation, and Peak Signal-to-Noise Ratio (PSNR). The HST model exhibited the best performance, with the lowest RMSE and highest correlation and PSNR values across almost all noise levels, as highlighted in red. The CNN and Transformer models follow closely, as marked in blue.

We then evaluated our method using experimental data. The experimental data used for the evaluation consist of laboratory experiments with 11 types of gases and field experiments with a blackbody and two gases. The gases in the laboratory experiments included Methane (CH_4_), Ethylene (C_2_H_4_), ammonia (NH_3_), sulfur hexafluoride (SF_6_), trimethylamine ((CH_3_)_3_N), Cyclopropane (C_3_H_6_), Propylene (C_3_H_6_), trans-2-butene (C_4_H_8_), butadiene (C_4_H_6_), Ethylene oxide (C_2_H_4_O), and vinyl chloride (C_2_H_3_Cl), while the gases in the field experiments included ammonia (NH_3_) and sulfur hexafluoride (SF_6_). In the laboratory experiments, a blackbody was used as an ideal background, and a customised gas chamber was placed between the blackbody and prototype with a 0.5 m optical path. Under these conditions, the measured gases were released from gas bottles into the gas cell. During the experiment, the background blackbody temperature was set at 50 °C. The gas temperature, humidity, and pressure in the gas cell were 23.1 °C, 40%, and 1 atm, respectively. The gas charged into the gas cell with 0.5%, 1%, and 2% purity, corresponding to path-integrated concentrations of 2500, 5000, and 10,000 ppm-m, respectively. The path integral concentration can be determined by multiplying the concentration with the gas cell’s optical path.

**Figure 5:**
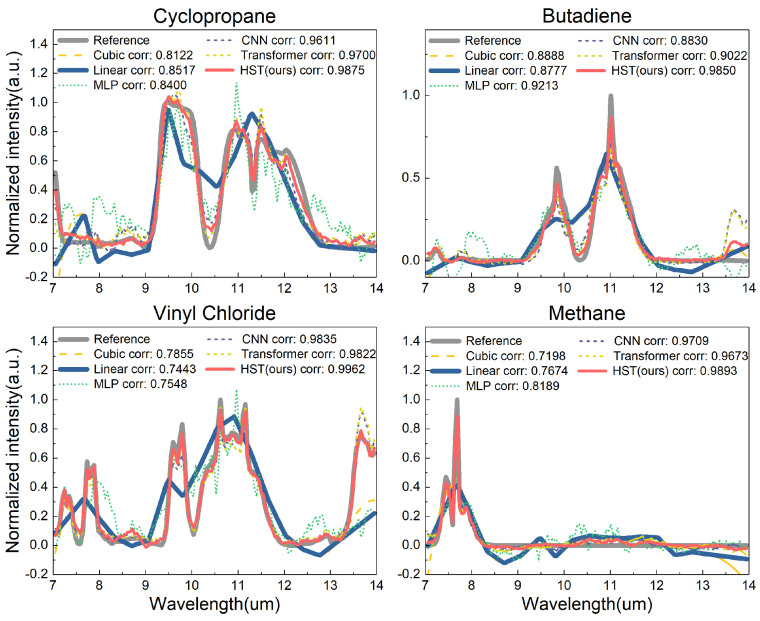
A comparison of different methods using experimental laboratory data of four different gas transmission spectra: Cyclopropane, Butadiene, vinyl chloride, and Methane. Each subplot shows the reference spectrum (in gray) alongside the spectra predicted by various models: Cubic Interpolation, Linear Interpolation, MLP, CNN, Transformer, and HST (ours). The correlation coefficients (corr) for each model are also provided, indicating the degree of similarity between the predicted and reference spectra. The HST model consistently shows the highest correlation across all molecules, suggesting superior performance in predicting the spectral features accurately.

We initially compared our proposed HST with other methods using laboratory experiments with gas transmission spectra. As illustrated in Figure 5, our proposed HST accurately reconstructed the high-resolution spectrum from the low-resolution input (Linear Interpolation, in solid blue line) for four different types of gas transmission spectra (Cyclopropane, Butadiene, vinyl chloride, and Methane) across a wavelength range of 7 to 14 μm. In comparison to the MLP, CNN, and Transformer methods, our proposed HST effectively captured the details that were lost by other methods, such as the peak at 9.5 μm for Cyclopropane. Overall, our proposed HST outperforms the baseline networks.

We also quantitatively evaluated the performance of different methods in Table 2. This table illustrates the performance of various interpolation and learning methods in terms of three metrics: Root Mean Square Error (RMSE), correlation, and Peak Signal-to-Noise Ratio (PSNR). The Linear and Cubic Interpolation methods show moderate performance, with RMSE values of 0.1149 and 0.1338, respectively. The MLP method offers a slight improvement with an RMSE of 0.1241 and a correlation of 0.9248. The CNN method significantly enhances the performance, achieving an RMSE of 0.0437 and a correlation of 0.9866. Among all the methods, the HST (ours) method demonstrates the best performance, with the lowest RMSE of 0.0333, the highest correlation of 0.9915, and the highest PSNR of 26.78, indicating its superior predictive capabilities. The second best performance is achieved by the Transformer method, marked in blue, with an RMSE of 0.0422 and a correlation of 0.9880. This table emphasizes the effectiveness of our HST method in accurately reconstructing the high-resolution spectrum.

**Figure 6:**
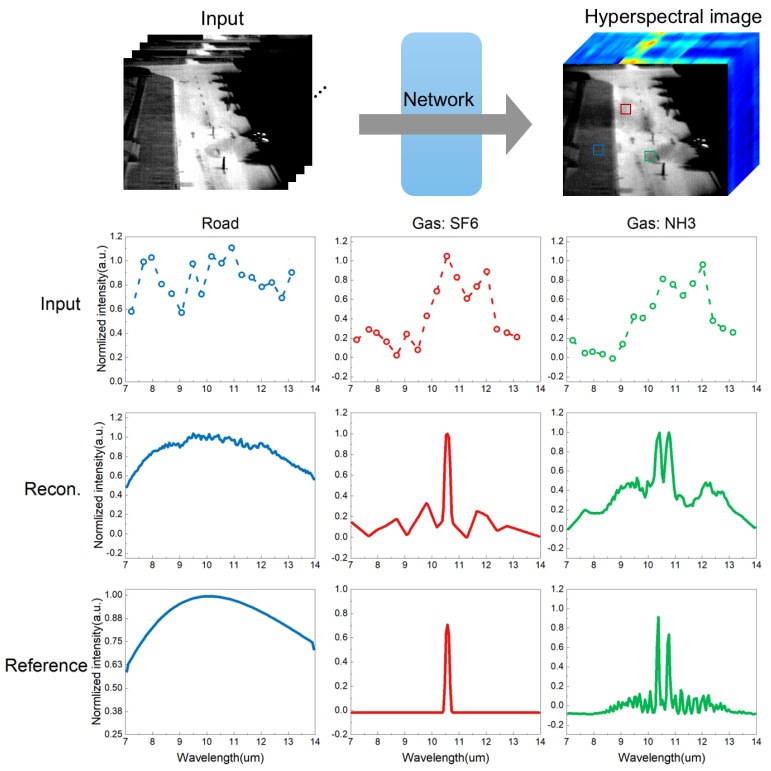
Illustration of HST using field experiments. The top row shows the input multi-spectral image, the network, and the reconstructed high-spectral resolution image. Below, three sets of graphs compare the input, reconstructed, and reference spectral data for three materials: a road, SF6 gas, and NH3 gas. The results highlight the network’s ability to accurately reconstruct high-resolution spectra.

Figure 6 illustrates the evaluation of our method using field experimental data. The experiment’s goal was to test the effectiveness of the spectral imaging in complex scenes. In an open field, we released SF6 and NH3 gases, each with a concentration of 40,000 ppm, marked by red and green boxes, respectively. The images were captured from approximately 100 m away and 15 m above ground. The ground’s temperature, around 35 °C, created a strong thermal contrast against the cooler gas temperature of 22 °C. This contrast helped highlight the gas targets. However, it also introduced significant noise into the raw data, alongside the inherent Poisson noise. Our HST was trained using synthetic data. We then used the HST to reconstruct the high-resolution spectrum from the noisy input intensity for each pixel. We selected three different targets for this process: a road, SF6 gas, and NH3 gas. The middle section of the figure offers a detailed comparison of the input, reconstructed, and reference spectral data for these three materials. The input data appear noisy and distorted when compared to the reference spectrum, primarily due to background and Poisson noise. However, the graphs in this section demonstrate the strong correlation between the input spectra and the network’s reconstruction against the reference spectrum. This emphasizes the network’s high accuracy in preserving spectral features. The displayed quantitative results further highlight the consistency and precision of our reconstructions.

## 4. Conclusions

The proposed Hierarchical Spectral Transformer presents an innovative solution to challenges in long-wave infrared spectral image reconstruction. By concentrating training on pixel-based LWIR spectra and incorporating an LWIR spectral noise model into the data simulation pipeline, the HST effectively bridges the gap between synthetic and experimental data, reducing the need for high-quality paired data. The multistage architecture of the HST provides a detailed and accurate reconstruction by progressively refining the spectral features and using spectral-wise multi-head self-attention to focus on relevant spectral features. This approach surpasses traditional CNNs, which struggle with noise and the unique characteristics of the LWIR spectrum. Despite the inherent challenge of the nature of the reconstruction problem, the HST demonstrates promising results in both synthetic and experimental data, indicating its potential for real-world applications in environmental monitoring, climate studies, and even carbon neutrality efforts.

## Figures and Tables

**Table 1:** Comparison of different methods with various noise levels using synthetic dataset.

	Noise Level	0	0.1	0.2	0.5
Linear interp.	RMSE	0.1133	0.1346	0.1793	0.3532
	Correlation	0.9558	0.9433	0.9134	0.8008
	PSNR	17.54	16.28	13.98	8.16
Cubic interp.	RMSE	0.1139	0.1686	0.2629	0.5953
	Correlation	0.9593	0.9180	0.8420	0.6868
	PSNR	17.93	14.33	10.13	2.75
MLP [32]	RMSE	0.1208	0.1130	0.1767	0.2153
	Correlation	0.9568	0.9599	0.9011	0.8420
	PSNR	18.04	18.35	14.63	12.83
CNN [26]	RMSE	0.0103	0.0233	0.0428	0.1076
	Correlation	0.9989	0.9968	0.9906	0.9565
	PSNR	33.87	29.22	24.53	18.03
Transformer [35]	RMSE	0.0061	0.0238	0.0436	0.1089
	Correlation	0.9994	0.9970	0.9904	0.9563
	PSNR	36.51	29.46	24.45	17.99
HST (ours)	RMSE	0.0059	0.0212	0.0378	0.1074
	Correlation	0.9995	0.9976	0.9929	0.9566
	PSNR	37.16	30.41	25.74	18.02

Red: the best performance. Blue: the second best performance.

**Table 2:** Quantitative comparison of different methods using experimental laboratory data. The HST method (ours) outperforms all the others, with the lowest RMSE, highest correlation, and highest PSNR, highlighting its superior accuracy in spectral data prediction. The second best performance is achieved by the Transformer method.

Method	Metric	Performance
Linear interp.	RMSE	0.1149
	Correlation	0.9386
	PSNR	18.30
Cubic interp.	RMSE	0.1338
	Correlation	0.9059
	PSNR	16.28
MLP [32]	RMSE	0.1241
	Correlation	0.9248
	PSNR	17.67
CNN [26]	RMSE	0.0437
	Correlation	0.9866
	PSNR	24.87
Transformer [35]	RMSE	0.0422
	Correlation	0.9880
	PSNR	25.32
HST (ours)	RMSE	0.0333
	Correlation	0.9915
	PSNR	26.78

Red: the best performance. Blue: the second best performance.

## Data Availability

The code and data are publicly avaiable at https://github.com/zeromakerplus/LWIR_spectral_reconstruction_public (accessed on 27 November 2024).

## Abbreviations

For the convenience of our readers, we have provided a table outlining the variables used in this paper, along with their meanings.

	**Greek Symbol**	**Meaning**	
	P	photon noise	
	τ	response	
	σ	variance	
	μ	mean	
	α	attention weight	
**Lowercase Label**	**Meaning**	**Uppercase Label**	**Meaning**
*t*	target	*L*	radiation
*p*	background	*M*	encoding dimension
*d*	dark current	*N*	noise
*q*	quantization	*Q*	query
*n*	total number of data	*K*	key
s	spectrum	*V*	value
g	ground truth spectrum	S	network input
l	length of spectrum	B	concatenated attention
